# Trophic support following peripheral axotomy show different behaviour of reactive microglia and astroglia in the ventral horn

**DOI:** 10.1186/2193-1801-4-S1-P34

**Published:** 2015-06-12

**Authors:** Michele Papa, Anna Maria Colangelo, Leonilde Savarese, Ciro De Luca, Giovanni Cirillo, Lilia Alberghina

**Affiliations:** Lab. of Morphology of Neuronal Network, Dept. of Public Medicine, Second University of Napoli, Italy; Lab. of Neuroscience “R. Levi-Montalcini”, Dept. of Biotechnology and Biosciences, University of Milano-Bicocca, Italy; SYSBIO, Centre of Systems Biology, University of Milano-Bicocca, Italy

**Keywords:** Gliopathy, motoneuron axotomy, NGF

Maintaining a neurotrophic support after peripheral nerve injury can be a key point in reducing the plastic changes which neurons, microglia, and astrocytes in the ventral horn undergo due to the loss of afferent synaptic and neurotrophic stimuli originating from the periphery. Therefore, intrathecal administration of trophic factors or the inhibition of the mechanisms responsible for their degradation could help prevent these changes. The purpose of our study was to analyze the changes in the ventral horn produced by gliopathy determined by the suffering of the motor neurons after peripheral nerve injury following spared nerve injury (SNI) of the sciatic nerve and how the administration of NGF or its synthetic analogue BB14, as well as the increase of endogenous NGF levels by i.t. infusion of GM6001, a MMPs inhibitor modulate these events. Immunohistochemical analysis of spinal cord sections revealed that SNI was associated with increased microglial (Iba1) and astrocytic (GFAP) responses, indicative of reactive gliosis. These changes were paralleled by (i) decreased glial aminoacid transporters GLT1 and GlyT1, and increased levels of neuronal glutamate transporter EAAC1, this maladaptive behavior of neuronal and glial EAATs is paralleled by a net increase of the Glutamate/GABA ratio as measured by HPLC analysis. These molecular changes were found to be linked to an alteration of endogenous NGF metabolism, as demonstrated by decreased levels of mature NGF. The continuous i.t. NGF infusion or of its analogue BB14, or of the generic MMPs inhibitor GM6001 reduced reactive astrogliosis and normalized the expression of neuronal and glial glutamate and glycine transporters, restoring the reduction of the Glutamate/GABA ratio but it showed to be absolutely ineffective in modifying the reactivity of microglia, demonstrating that the two glial populations have different mechanisms of modulation associate to neuronal damage.

